# Characterization of Different Molecular Size Fractions of Glomalin-Related Soil Protein From Forest Soil and Their Interaction With Phenanthrene

**DOI:** 10.3389/fmicb.2021.822831

**Published:** 2022-02-23

**Authors:** Xian Zhou, Jian Wang, Yi Jiang, Ganghua Leng, Galina K. Vasilyeva, Michael Gatheru Waigi, Yanzheng Gao

**Affiliations:** ^1^College of Resources and Environmental Sciences, Institute of Organic Contaminant Control and Soil Remediation, Nanjing Agricultural University, Nanjing, China; ^2^Institute of Physicochemical and Biological Problems in Soil Science, Russian Academy of Sciences, Moscow, Russia

**Keywords:** glomalin-related soil protein (GRSP), molecular weight, spectroscopic characterization, phenanthrene, association coefficient

## Abstract

As a natural organic compound secreted by arbuscular mycorrhizal fungi (AMF), glomalin-related soil protein (GRSP) is an important part in soil, affecting the bioavailability of polycyclic aromatic hydrocarbons (PAHs) in it. Previous research have demonstrated that GRSP could enhance the availability of PAHs in the soil and favor their accumulation in plant roots. However, a scarcity of research exists on the different molecular weights of GRSP interacting with PAHs due to their complexation and heterogeneity. In this research, the extracted GRSP in soil was divided into three molecular weight (Mw) fractions of GRSP (<3,000, 3,000–10,000, and >10,000 Da), whose characteristics and binding capacity of PAHs were conducted by using UV–visible absorption, quenching fluorometry and, Fourier transform infrared spectroscopy. The results showed that the GRSP was composed of abundant compounds, it has a wide distribution of molecular weight, and the >10,000 Da Mw fraction was dominant. For three Mw fractions of GRSP, they have some difference in spectral features, for example, the >10,000 Da fraction showed higher dissolved organic carbon (DOC) contents, more phenolic hydroxyl groups, and stronger UV adsorption capacity than the low and middle Mw fractions. In addition, the interaction between GRSP and phenanthrene is related to the characteristics of the Mw fractions, especially the phenolic hydroxyl group, which has a significantly positive correlation with a binding coefficient of *K*_*A*_ (*k* = 0.992, *p* < 0.01). Simultaneously, hydrophobic, NH-π, and H-bound also played roles in the complexation of phenanthrene with GRSP. These findings suggested that different GRSP_*Mw*_ fractions could influence the fate, availability, and toxicity of PAHs in soil by their interaction.

## Introduction

The terrigenous organic matter plays a vital role in controlling the ibogeochemical processes of contaminants, such as heavy metals and organic contaminants (Wang et al., 2020). Enormous reports have indicated that soil organic matter (SOM), like dissolved organic matter (DOM), can increase the solubility of toxic pollutants, enhance their transfer and accessibility in soil, exacerbate the environmental risks of pollutants, etc (Hur et al., 2014; Lin et al., 2018). However, a great proportion of environmental functions for pollutants in terrestrial organisms, such as fungi and their secretions with symbiotic plants, remains unclear.

As a secretion of arbuscular mycorrhizal fungi (AMF) in soil, the enormous amount of GRSP in the soil is desirable not only to quantify AMF potential for carbon sequestration but also to improve water-stable aggregates in soil and influence soil fertility (Wright and Upadhyaya, 1996, 1998; Borie et al., 2006; Nichols and Wright, 2006), and so it may remedy soil contamination by complexing with potentially toxic elements, such as heavy metal or organic pollutants in soil (Wang et al., 2020; Tian et al., 2021). To explore the environmental function of GRSP, it was divided into total glomalin-related soil protein (T-GRSP), easily extractable glomalin-related soil protein (EE-GRSP), immunoreactive total glomalin-related soil protein, and immunoreactive easily extractable glomalin-related soil protein (Rillig, 2004). Chi et al. (2018) found that exogenous EE-GRSP could improve the drought tolerance of trifoliate. Gao et al. (2017) found that EE-GRSP and T-GRSP could influence the sorption processes of organic pollutants, like PAHs, in soil and enhance the accumulation of PAHs in roots (Chen et al., 2018). However, many studies have reported that soil-derived GRSP contains some heat-stable proteins, lipids, and humic substances of non-AM fungi (Schindler et al., 2007; Gillespie et al., 2011), which restricted the research on GRSP. Furthermore, some researchers observed that GRSPs have a wide molecular size distribution (Wright and Upadhyaya, 1996, 1998; Bolliger et al., 2008; Gillespie et al., 2011). Nevertheless, there is a scarcity of reports on the different molecular weights of GRSP, which may have different functional groups.

As various carcinogenic and mutagenic, persistent, organic, and health-threatening pollutants (Wang et al., 2017), the environmental behavior (e.g., transportation and bioavailability) of polycyclic aromatic hydrocarbon (PAHs) in soil is primarily affected by SOM. Extensive studies have reported that sediments, organic carbon, humic acids, and suspended particles can strongly bind with PAHs (Pan et al., 2007; Wu and Sun, 2012; Hur et al., 2014). At the same time, in our previous studies, we showed that the GRSP could influence the sorption processes of organic pollutants, like PAHs, in soil (Gao et al., 2017; Chen et al., 2019). However, there is no evidence to indicate the binding process between PAHs and GRSP.

In this study, GRSP was firstly divided into components of different molecular weight (Mw), according to Lin et al. (2018), such as <3,000, 3,000–10,000, >10,000 Da, and GRSP bulk fraction, aimed at elucidating the impact of the chemical composition of GRSP on its binding for PAHs by studying phenanthrene (as a model of PAHs) binding onto different Mw fractions of a field-extracted GRSP sample. Spectroscopy and mass spectrometry instruments, such as UV–visible spectroscopy, GC/MS, and Fourier transform infrared (FTIR), were applied to analyze the composition and structure of GRSP. Fluorescence quenching experiments of phenanthrene by different Mw fractions were conducted. We also explored the relationship between different Mw fractions of GRSP and phenanthrene to illustrate the underlying roles and mechanisms of GRSP on PAHs. The findings herein would broaden our knowledge of the transport and fate of PAHs in soils and the unique contents of GRSP.

## Materials and Methods

### Test Chemicals

Phenanthrene (solid powder) with purity of >98% was acquired from Sigma-Aldrich Fluka (United States). Stock solutions of the phenanthrene standard were prepared by dissolving 1 mg/ml in methanol, which was stored in a volumetric flask at −4°C in the dark and used for the preparation of fluorescence quenching experiments. A fresh stock solution was prepared monthly. All organic reagents (HPLC grade) and inorganic reagents (analytical grade) were obtained from Nanjing Chemical Reagents Company (Nanjing, China). Sterile ultrapure water (18 MΩ × cm) was used throughout the experiments.

**FIGURE 1:**
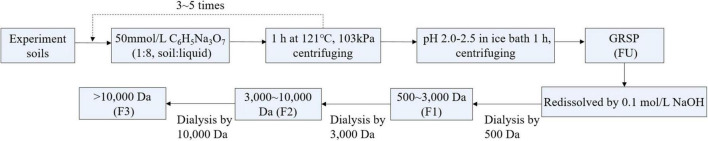
Sequential extraction of glomalin-related soil protein with a different molecular weight in soil.

In a previous study, we found that the concentration of GRSP in forest soil is apparently higher than that in other land utilization types (Que et al., 2015). Therefore, T-GRSP was extracted in forest soil from Purple Mountain in Nanjing, Jiangsu Province, China. The properties of the forest soil are listed in Supplementary Table 1. The GRSP extractions were determined based on the method described by Wright and Upadhyaya (1996), with minor modifications (Figure 1), and the detailed steps of preparation are introduced in the Supplementary Material.

### Fractionation and Characterization of Glomalin-Related Soil Protein

A part of the prepared GRSP solution, obtained through the thermal sodium citrate method, was divided into three size fractions by ultrafiltration centrifugal tubes (Merk America) containing ultrafiltration membranes with nominal molecular weight cutoffs of 10,000 and 3,000 Da, which were expressed as <3,000 Da (F1), 3,000–10,000 Da (F2), and >10,000 Da (F3), and the unfractionated GRSP was expressed as FU (Supplementary Figure 1); this method referred to Lin et al. (2018). The ultrafiltration procedure details are shown in the Supplementary Material. Next, the GRSP concentrations were detected by Bradford assay using bovine serum albumin as the standard (Wright and Upadhyaya, 1998; Table 1) and stored at 4°C before use. Gel permeation chromatography (GPC; Waters 1525, United States) was used to analyze the molecular weight sizes of the GRSP fractions that were characterized (Supplementary Figure 2).

**TABLE 1:** The characteristics of GRSP and its different MW fractions.

GRSP fraction	GRSP	DOC	Fraction mass (%)					Atomic ratios				Carboxylic acids		Phenolic hydroxyl group		Total acidity
	(mg/L)	(mgC/L)	C	N	H	S	O	C/H	C/N	O/C	(N+O)/C	Concentration (mol/kg)	p*K*a	Concentration (mol/kg)	p*K*a	
F1	10.60	440.52	23.99	0.08	3.22	0.22	44.76	7.45	300	1.87	1.87	8.44	5.56	0.63	9.5	9.07
F2	3.87	110.81	17.67	0.36	3.43	0.20	40.02	5.15	49.08	2.26	2.29	7.99	5.42	0.35	9.62	8.34
F3	23.57	998.97	12.57	1.09	2.86	0.16	24.51	4.39	11.53	1.95	2.04	5.31	5.5	0.94	8.14	6.25
FU	21.37	939.68	14.02	1.17	3.22	0.11	25.04	4.35	11.98	1.78	1.87	5.63	5.03	1.88	8.37	7.51

*F1–F3 represents different molecular fractions from <3,000, 3,000–10,000, and >10,000 Da, respectively. DOC, dissolved organic carbon; FU, GRSP bulk.*

The multi N/C 3100 (Jena, Germany) was used to quantify the DOC concentrations of GRSP fractions. The specific ultraviolet absorbance (SUVA) of compounds were determined by a UV–visible spectrometer (UV7200, Shimadzu, Japan) with 1 cm cuvette in order to calculate the SUVA_254_, SUVA_260_, and SUVA_280_ values. The elements of GRSPs were analyzed by Elementar Vario EL cube, which was made in Germany. At the same time, we attempted to break the ester and ether bonds of GRSP by using the method from Chen et al. (2021) to analyze the molecular composition. The pyrolysis method details are shown in the Supplementary Material.

### Fluorescence Quenching Experiments

Phenanthrene was used as a model PAH for this study because of its wide distribution in polluted soils. A fluorescence quenching technique was adopted to determine the interaction between phenanthrene and fractions of GRSP. This method has already been used in most studies examining variations in the *K*_*OC*_ values of different DOM samples (Wu and Sun, 2012; Zhao et al., 2019) and been proven for reliability.

Then, 5 ml of phenanthrene solution (less than 0.1% methanol) was spiked into each GRSP solution (0 to 1 mg/L). The added phenanthrene concentration in each glass bottle was 0.5 mg/L, which was within the solubility of phenanthrene. The samples were equilibrated in bottles on a horizontal shaker (200 rpm, 25°C, 30 min). After reaching equilibrium, the fluorescence intensities were recorded on a fluorescence spectrophotometer (F97 pro, Shanghai Kehuai Instrument Co., Ltd., China) with a quartzose cuvette (1 cm, rectangular) at the excitation (*E*_*x*_)/emission (*E*_*m*_) wavelengths of 292/365 nm. Ultrapure water was used as a blank.

The obtained data was used to calculate the quenching constant (*K*_*sv*_), the associated constant (*K*_*A*_), the number of binding sites (*n*) with the Stern–Volmer equation (Ferrie et al., 2017), and the site binding equation, respectively (Bai et al., 2017).

The Stern–Volmer equation is listed as Eq. (1):


(1) $$\frac{F_{0}}{F}=1+K_{\text{SV}}\,\left[\text{GRSP}\right]$$

Where *F*_0_ is the original phenanthrene fluorescence intensity, and *F* is fluorescence degrees after the addition of quencher agents (GRSP). In this study, GRSPs do not possess intrinsic fluorescence at fluorescent regions of phenanthrene; therefore, we did not remove the background effects. [GRSP] is the concentration of GRSP.

The site binding equation is shown as Eq. (2):


(2) $$\log\frac{F_{0}-F}{F}=\log K_{\text{A}}+n\,\log\left[\text{GRSP}\right]$$

### Fourier Transform-Infrared Measurement

The phenanthrene–GRSP mixtures at high concentrations were prepared in order to acquire better infrared spectra before freeze-drying. The obtained particles and GRSP particles were ground and formed into tablets with the spectrographic grade of KCl, respectively. All spectra were measured with a Nicolet Nexus 870 FT-IR spectrometer (Thermo Fisher Scientific Co., Ltd., United States) at 400–4,000 cm^–1^.

### Statistical Analyses

Pearson correlation analysis (SPSS, United States) was used to select related properties of the GRSP fractions and their *K*_*sv*_ and log*K*_*A*_. All the statistics were executed using Microsoft Excel (Microsoft, Redmond, Washington, DC, United States), while Origin software (OriginLab, Northampton, MA, United States) was used to make the figures.

## Results

### Fractionation of Glomalin-Related Soil Protein

The distribution of GRSP molecular weight was obtained by GPC. As shown in Supplementary Figure 2 and Supplementary Table 2, the GRSP had a wide molecular weight distribution because of its broad distribution and higher polydispersity (PDI = 2.40). The distribution of measured apparent molecular weights ranged from 923 to 88,311 Da. According to the main peak of the GPC graph and standard curves (Supplementary Figure 2A), the average apparent molecular weight was calculated and summarized in Supplementary Figure 2C. The apparent molecular weight (Mw), numerical molecular average weight (Mn), and peak molecular weight (Mp) were 19,247, 8,018, and 16,933 Da, respectively. Furthermore, the molecular weight fractions of the dark-colored GRSP sample (FU) were divided into light-yellow-colored F1 fraction (<3,000 Da), faint-yellow-colored F2 fraction (3,000–10,000 Da), and dark-brown-colored F3 fraction (>10,000 Da) (Supplementary Figure 1). F3 is the main component of GRSP (Supplementary Figure 2B), which has a proportion of 63%. F1 and F2 accounted for 10 and 27%, respectively.

The GRSP cracking information is offered in Supplementary Table 3. The KOH–MeOH, at 100°C for 3 h, can break the chemical bond (e.g., ester bond and ether bond) of SOM and release molecular from heterogeneous SOM (Nebbioso and Piccolo, 2011; Chen et al., 2021). As shown in Supplementary Table 3, most fatty acids, esters, and phenols/lignin monomers were divided by GC/MS/MS, suggesting that GRSP is a high molecular weight aggregate formed by the bonding of numerous small organic molecules. Here, we gained fewer protein/peptide signals (only eight amino compounds in 48 main products) and more abundant signals for carbohydrates depended on this method.

### Comparison of Characteristics for Different Molecular Fractions of Glomalin-Related Soil Protein

#### Elements

The elemental analysis results verified that there are significant differences in element composition among GRSP components with different molecular weights (Table 1). The C and O contents in F3 were lower than F1 and F2, and the H contents were the same in the three fractions of GRSP, whereas the N contents were larger with an increase in molecular weight, the percentage of which increased from 0.06% to 1.09%. On the side, atomic ratios could reflect some structure information on GRSP. For F3, a higher O/C showed that there are more oxygenic groups (like carboxylic acids), and its structure has scattered porosity. A larger C/N ratio of F1 meant that it has higher stability than the other fractions.

#### Potentiometric Titration

In order to evaluate the complexation of GRSP, it is necessary to understand the dissociation characteristics of acidic functional groups (Wu and Sun, 2012). For this study, equivalent NaOH represented a charge density. As shown in Table 1 and Supplementary Figure 3, the p*K*a values of the carboxyl and phenolic hydroxyl groups in GRSP were about 5.03 and 8.37, respectively. With an increase in molecular weight, the carboxylic acid concentration decreased from 8.44 to 5.31 mol/kg; the concentration of phenolic hydroxyl groups increased from 0.63 to 0.94 mol/kg, except the F2 fraction whose phenolic hydroxyl group content was only 0.35 mol/kg. Similarly, the differences of total acidity from F1 to F3 were apparent, such that, with an increase in molecular weight, the total acidity was decreased.

#### UV–Visible Spectrum

**FIGURE 2:**
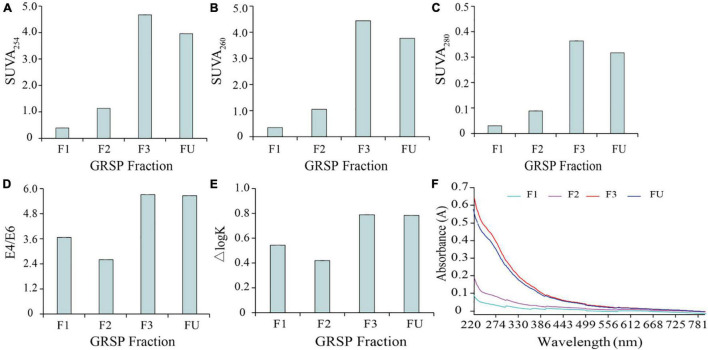
UV–visible spectra of glomalin-related soil protein (GRSP)_*MW*_ solution. The SUVA_254_ of GRSP_*MW*_ solution **(A)**, SUVA_260_ **(B)**, and SUVA_280_ **(C)** express the degree of aromatization and hydrophobicity of GRSP_*MW*_, respectively; E4/E6 means the ratio of UV being 465 and 665 nm **(D)**, which characterized the degree of decay and molecular weight of humus; Δlog*K* is the tonal coefficient, the logarithm difference between the absorbance at 400 and 600 nm **(E)**. **(F)** Absorbance of GRSP_*MW*_ from 220 to 800 nm. FU, F1–F3 represents different molecular fractions from GRSP_*bulk*_: <3,000, 3,000–10,000 and 10,000 Da, respectively.

The values of SUVA_254_, SUVA_260_, Δlog*K* (tonal coefficient, which meant the logarithm difference value between 400 and 600 nm), and E4/E6 (E465/E665) are always presented as an index to estimate the aromatic concentration in DOM, hydrophobic property, and oxidation level of DOM (Weishaar et al., 2003). In the present study, these values of the GRSP fractions, with respect to the different molecular weights, were compared (Figure 2). With increases in molecular weights, the SUVA_254_, SUVA_260_, and SUVA_280_ values significantly increased from 0.39, 0.35, and 0.03 to 4.67, 4.44, and 0.36, respectively (Figures 2A–C), indicating the evidently complex changes of GRSP structures. The aromatic ring condensation, aromatization, and molecular weight of GRSP were reflected, as shown in Figure 2D. The E4/E6 value of F2 was lower than the other GRSP fractions, which resulted in lower aromaticity. Figure 2E showed that a sizeable molecular weight has higher Δlog*K*, suggesting that the high molecular weight of GRSP has a low content of carboxyl and phenolic hydroxyl groups and a low degree of oxidation. In addition, the UV–visible spectra of GRSP Mw have been drawn and are shown in Figure 2F. Every GRSP fraction has an intense light absorption capacity from 220 to 400 nm, and the absorption of GRSPs has an exponential decrease. In a range from 220 to 650 nm, a higher molecular GRSP has stronger UV absorption than a low molecular weight.

### The Binding Properties of Glomalin-Related Soil Protein on Phenanthrene

**FIGURE 3:**
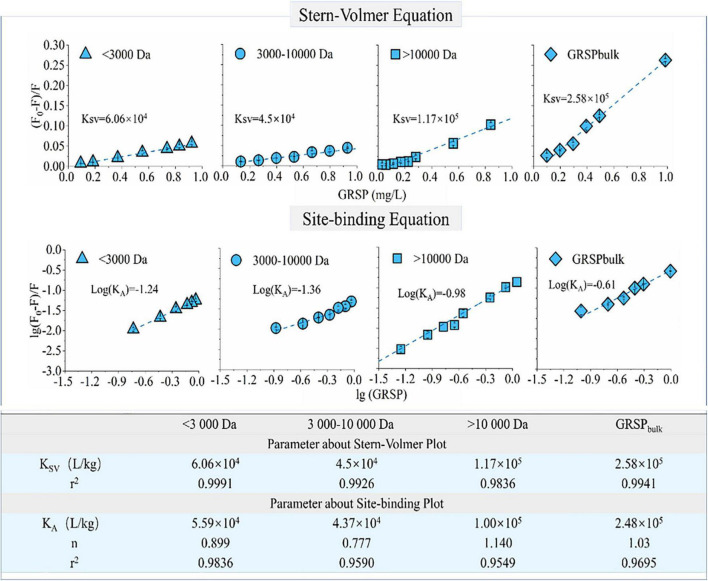
Phenanthrene-glomalin-related soil protein (GRSP) binding probed by GRSP-caused fluorescence quenching. The quenching constant (*K*_*sv*_), binding constant (*K*_*A*_), and number of binding sites (*n*) for the binding of phenanthrene with GRSP were calculated from the Stern–Volmer plot and site-binding plot, respectively.

The binding ability of phenanthrene to components of GRSP with different molecular weights was significantly different. The fluorescence quenching curves of phenanthrene and GRSP with different concentrations have a good linear relationship, in which *r*^2^ was from 0.9836 to 0.9991 (Figure 3). As shown in Figure 3, the GRSP binding constant *K*_*A*_ was close to the quenching constant *K*_*sv*_, suggesting that the quenching processes were static. The quenching constant between GRSP _>__10,000__*Da*_ and phenanthrene was higher than the others. The K_*GRSP*__>__10,000_ (site binding constant between phenanthrene and GRSP _>__10,000_) was 1.00 × 10^5^ L/kg, which was twice as large as *K*_*GRSP*__< 3,000_ and *K*_*GRSP3,000*–10,000_. The site binding values of GRSP _< 3,000_ and GRSP_3,000–10,000_ were 5.59 × 10^4^ and 4.32 × 10^4^ L/kg, respectively. In addition, for every GRSP fraction, the binding site number *n* was near 1.00, indicating that the binding of GRSP and phenanthrene belongs to a monomolecular binding nature.

The thermodynamic experiment presented that the binding processes were stronger at higher temperatures. The corresponding values of ΔH^0^, ΔS^0^, and ΔG^0^ were obtained in Supplementary Table 4. A positive ΔH^0^ for GRSP–phenanthrene complexes showed that the binding reaction was endothermic. Meanwhile, a positive ΔS^0^ suggested that the conformation arrangement of phenanthrene and GRSP would be unordered (Xu et al., 2013). The negative ΔG^0^ indicates that the binding processes between phenanthrene and GRSP were spontaneous. | ΔG^0^_*F1*_| > | ΔG^0^_*F2*_| > | ΔG^0^_*F3*_| at the same temperature condition, which verified that the GRSP _>__10_,_000__*Da*_ fraction had the largest binding capability with phenanthrene.

Different pH and ionic strengths could induce alterations of the GRSP interaction with phenanthrene. The association constant of GRSP–phenanthrene fluctuated when the pH values ranged from 3.0 to 11.0 at ionic strength 2 mmol/L (Supplementary Figure 5). For the GRSP _< 3,000_–phenanthrene system, the *K*_*A*_ was decreased from 0.065 to 0.047 L/mg with increased pH. At pH from 5 to 11, a higher binding capacity of GRSP_3,000–10,000_ was obtained. However, for GRSP and GRSP _>__10,000_ fractions, the alterations of pH seem to have less influence on the binding between GRSP and phenanthrene. Moreover, the high ionic strengths and cationic type could result in weak binding in the GRSP–phenanthrene systems, as shown in Supplementary Figure 4 and Supplementary Table 5. Otherwise, the F2–phenanthrene system did not affect the binding ability with the increase of cationic valence.

### The Interaction Mechanism Between Glomalin-Related Soil Protein and Phenanthrene

For this study, we found that the binding of GRSP and phenanthrene had a significant correlation between phenol hydroxyl, S (Table 2). Among these parameters, the phenolic hydroxyl groups had a significantly high positive correlation with *K*_*sv*_, with *K* values of 0.994. Table 2 shows that with more phenolic hydroxyl, a higher binding capacity between phenanthrene and GRSP occurred, wherein the *K* value was 0.992 (*p*-value < 0.01).

**TABLE 2:** Bivariate correlations between GRSPs characteristics and *K*_*sv*_/*K*_*A*_.

	*K*_*sv*_	*P*-value	log*K*_*A*_	*P*-value
DOC	0.523	0.477	0.625	0.375
SUVA254	0.694	0.306	0.764	0.236
Total acidity	–0.43	0.57	–0.519	0.481
Carboxylic acids	–0.738	0.262	–0.804	0.196
Phenolic hydroxyl group	0.994^b^	0.006	0.992^b^	0.008
O/C	–0.692	0.308	–0.74	0.26
(N O)/C	–0.556	0.444	–0.596	0.404
C/H	–0.578	0.422	–0.585	0.415
C/N	–0.493	0.507	–0.49	0.51
SUV260	0.696	0.304	0.766	0.234
ΔlogK	0.782	0.218	0.864	0.136
SUV280	0.71	0.29	0.778	0.222
E4/E6(E465/E665)	0.78	0.22	0.862	0.138
C	–0.583	0.417	–0.619	0.381
N	0.804	0.196	0.847	0.153
H	–0.213	0.787	–0.352	0.648
S	−0.954^a^	0.046	−0.953^a^	0.047
O	–0.763	0.237	–0.817	0.183

*^a^Significant difference between treatments at 0.05 level. ^b^Significant difference between treatments at 0.01 level.*

**FIGURE 4:**
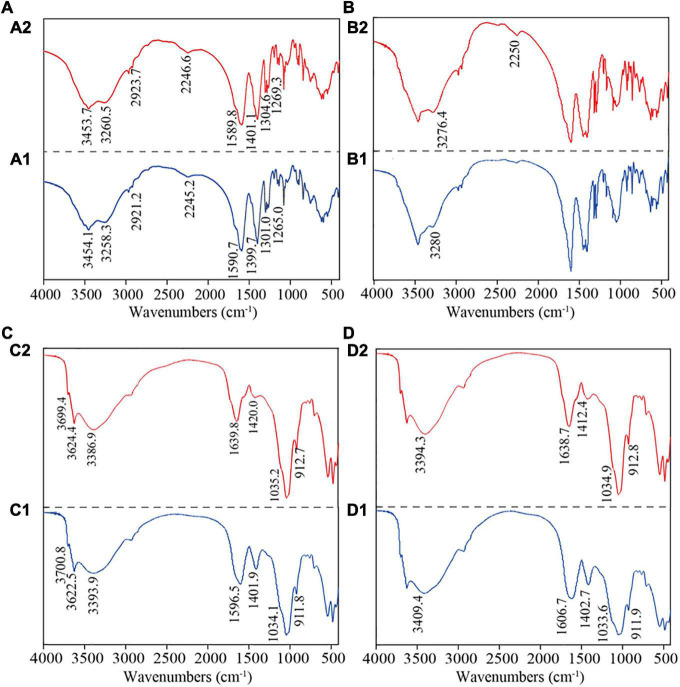
The FTIR analysis of interactions between phenanthrene and molecular weight glomalin-related soil protein (GRSP). **(A1–D1)** means the functional groups of GRSP _< 3,000__*Da*_, GRSP_3,000–10,000__*Da*_, GRSP _> 10,000__*Da*_, and GRSP_*bulk*_. **(A2–D2)** means the changes after the interaction between GRSP and phenanthrene.

The fractions of GRSP responsible for phenanthrene interaction and the corresponding binding capacity were determined based on FTIR analyses (Figure 4). The alteration in peak frequency of the FTIR spectra of phenanthrene-loaded GRSP fractions manifested that active functional groups (e.g., C = O, C–N, O–H, and –COO-) were involved in phenanthrene sequestration (Figure 4). Notably, the offset of functional groups (O–H stretching, C = O stretching associated with proteins, and C = O symmetric stretching) was larger when GRSPs adsorbed phenanthrene. In addition, the spectra of phenanthrene-sequestrated GRSP_*Mw*_ were significantly different, particularly for low molecular weight GRSP, while the variations of the characteristic peak were not significant.

## Discussion

### Characteristics of Glomalin-Related Soil Protein

Environmental factors, including land use, soil characteristics, and climatic conditions, have effects on the GRSP contents and compositions (Schindler et al., 2007; Zhang et al., 2015; Kumar et al., 2018). Therefore, the chemical structure of GRSP extracted in soil is mysterious and generally complex.

Under repeated autoclaving of soil in citrate buffer, GRSP extracted in soil contained various proteins, humic acids, and other compounds (Driver et al., 2005; Gillespie et al., 2011). The gross of GRSP can be characterized by GPC and GC/MS at the atomic and molecular scales. This information is beneficial toward determining the classes of compounds released by extraction procedures to provide assessments of the bulk composition of GRSP. For GPC analysis, the GRSP in forest soil ranged from 0.92 to 88.31 kDa, and the average molecular weight was at 17.0 kDa. Among them, >10 kDa molecular weights were major parts, accounting for 67% in GRSP bulk. However, some researchers also observed that some GRSPs in soil or AMF fungal hyphae have a higher molecular weight, in the bands between 55 and 65 kDa, between 60 and 90 kDa, or between 90 and 100 kDa, by using the SDS-PAGE method (Wright and Upadhyaya, 1996, 1998; Bolliger et al., 2008; Gillespie et al., 2011). These suggested that soil characterizations or varying extractant procedures cause different GRSP components in the soil to be released further.

For this study, the amount of GRSP fractions extracted from the forest soil was much lower than the range of GRSPs and its protein content previously reported for tropical and mineral soils, which operationally defined that glomalin reportedly contains 28–45% C, 0.9–7.3% N, and 0.03–0.1% P (Schindler et al., 2007; Sousa et al., 2012; Wang et al., 2014). However, the O contents of GRSP in this study were consistent with those reported by Schindler et al. (2007). This phenomenon could be associated with the C and N contents of forest soil.

As shown in the UV spectrometry, the high absorbance of GRSP_*bulk*_ and GRSP _>__10,000__*Da*_ is near 220 nm, which initiated the peptide bond. From 200 to 300 nm, the absorbance of GRSP lower than the Mw decreased. This range is the absorption band generated by the conjugated double bond π-π transition and is the characteristic absorption band of the aromatic ring and aromatic heterocyclic compound (Wang et al., 2014). So far, there are few studies on GRSP characteristics depending on the UV or fluorescent spectrometry. However, these values could show more structural information on GRSP. In brief, the aromaticity and hydrophobicity of the GRSP fractions with different molecular weights (Mw) were significantly different. High-Mw GRSP fractions had strong hydrophobicity, which easily bound with hydrophobic organic pollutants, thus forming complexes. Furthermore, Δlog*K* is inversely proportional to the content of the carboxyl group in DOM composition and is directly proportional to the molecular weight. These results obtained are consistent with potentiometric titration.

Studies have reported that there are abundant functional groups in GRSPs, such as O–H, N–H, C = O, – COO-, and C–N (Wang et al., 2014; Zhang et al., 2017). This was similar with our results (Figure 4). However, for fractions of GRSPs with different Mw values, their composition characteristics were not determined. In the present study, the composition characteristics of the three GRSP fractions with different molecular weights that we tested have some differences, especially between the >10,000 and 3,000-10,000, and <3,000 Da fractions (Figure 4), for instance, the absorption peaks of GRSP _>__10,000__*Da*_ at 1,600–500 cm^–1^ were weaker in intensity and narrower than the other fractions (Figure 4). GRSP is an integral part of dissolved soil organic matter, and its structural characteristics are similar to DOM (Schindler et al., 2007). Those at 4,000–3,000 cm^–1^ were associated with hydrocarbons, those at 1,800–1,200 cm^–1^ were associated with proteins, those at 1,200–1,000 cm^–1^ were associated with polysaccharides, and those at 1,000–800 cm^–1^ were related to nucleic acids (Yin et al., 2015). These infrared spectra suggested that the functional group GRSP_*bulk*_ is related to macromolecular substances, and the results in our study were in accordance with recent studies which reported the GRSPs to be a mixture of compounds (e.g., proteins, humic acids) (Schindler et al., 2007; Gillespie et al., 2011; Holátko et al., 2021). Furthermore, the GC/MS results also suggested that GRSPs were a complex mixture including organic acids and phenols in the soil (Supplementary Table 3).

### The Association Between Glomalin-Related Soil Protein and Phenanthrene

In the early 1970s, the experimental determination of binding coefficients (*K*_*DOC*_) of hydrophobic organic compounds (HOCs) has become a subject of intense interest (Krop et al., 2001). The present study showed a higher binding capacity between GRSP and phenanthrene, whose *K*_*A*_ value reached 2.48 × 10^5^ L/kg; the binding site number was 1.03. The binding coefficients in our study are beyond the ranges reported in other prior studies related to SOM binding with PAHs, for example, with the fluorescence quenching technique to follow anthracene binding to various humic materials, *K*_*OC*_ values varied from 1 × 10^4^ to 6 × 10^4^ L/kg (Gauthier et al., 1986). Hur et al. (2014) found a high adsorption affinity from phenanthrene interaction with terrestrial DOM in sediment surfaces; the *K*_*OC*_ values ranged from 3.6 × 10^3^ to 2.5 × 10^4^ L/kg. Compared with other SOM materials, Yang et al. (2011) determined that the ranges of *K*_*DOC*_ values between phenanthrene and dissolved humic acids were between 1.3 × 10^4^ and 2.5 × 10^4^ L/kg. DOM of higher ages have a strong binding capacity with phenanthrene; its *K*_*OC*_ value was 1.02 × 10^5^ L/kg (Xi et al., 2012). Our observed *K*_*A*_ values were higher than those values reported. The phenomenon indicated that the chemical and structural nature of GRSP greatly differed from terrestrial DOM. At the same time, a linear relationship between GRSP and phenanthrene was obtained by using the Stern–Volmer equation at different GRSP concentrations (0–1 mg GRSP/L). On the contrary, Chen et al. (2019) obtained the distribution coefficients between GRSP and phenanthrene using the Freundlich equation, which was consistent with our results. At the same time, Chen et al. (2019) suggested that a higher concentration could inhibit the interaction between phenanthrene and GRSP. This phenomenon may result from conformational and structural changes at higher GRSP concentrations and with different assessment methods. Additionally, as a complex and heterogeneous mixture, the GRSP interaction with HOCs is closely related to the molecular weight distribution of GRSP. In our study, with the fluorescence quenching technique, to follow phenanthrene binding to GRSP materials of different Mw values, the *K*_*A*_ values varied from 4.37 × 10^4^ to 1.00 × 10^5^ L/kg, and those with higher Mw fractions (>10,000 Da) have strong binding affinities; the binding coefficient is 1.00 × 10^5^ L/kg. The result is similar to that of Lin et al. (2018). The *K*_*DOC*_ of pyrene in DOM fractions of different molecular weights was ordered as high-Mw fractions > middle-Mw fractions > low-Mw fractions. The *K*_*DOC*_ of pyrene in the high-Mw-fraction DOM solution was up to 1.84 × 10^5^ L/kg.

### The Mechanism on the Binding Affinity Between Glomalin-Related Soil Protein and Phenanthrene

Recent studies have indicated that DOMs can bind numerous HOCs, such as PAHs, antibiotics, and pesticides (Wu and Sun, 2012; Zhao et al., 2019). Usually, the interaction between DOM and HOCs is mainly attributed to hydrophobic interactions, Van der Waals force, and H-bonding. In parallel, the π-π conjugate stacking effect and the electrostatic effect should be considered (He et al., 2009). However, the phenanthrene binding mechanisms with GRSP fractions of different Mw remain largely unknown. According to the changes of ΔS^0^ and ΔH^0^ in the bonding process (Supplementary Table 4), the force between phenanthrene and GRSP seems to be mainly a hydrophobic interaction because ΔS^0^ > 0 and ΔH^0^ > 0. The entropy change caused the complex formation due to | ΔH| < | TΔS|, which is in agreement with the results on the interaction of fluoroquinolones (FQs) with humic acid (HA) (Zhao et al., 2019).

Almost all studies on the binding between DOM and HOCs showed that their interaction is an ideal monomolecular binding mode (Pan et al., 2007; Zhao et al., 2019); this finding conformed with our study. The binding mode between phenanthrene and GRSP was monomolecular, and the *n* values were 0.777–1.140 with the Mw changes. In our investigation, from FT-IR spectra, we found that the absorption intensity of infrared absorptions was weakened and narrower at 3,700–3,400 cm^–1^ (-OH stretching), 1,700–1,600 cm^–1^ (amide I, C = O, and –COO- stretches), and 1,500–1,300 cm^–1^ (amide II, – COO-, and C = O stretching) when phenanthrene was loaded with GRSP (Figure 4). The functional groups at 1,800–1,200 cm^–1^ are associated with proteins, according to Yin et al. (2015), indicating that GRSP-bound phenanthrene was linked with proteins. Moreover, the peaks at 1,606.7 and 1,402.7 cm^–1^ drifted in the long-wavelength direction, while the peak at 3,409.4 cm^–1^ drifted in the short-wavelength direction (Figure 4D). Zhao et al. (2019) proved that H-bonding was involved in the FQs–HA complex due to the peak of –OH of FQs shifting to a low wave number in HA systems. That is to say, in the present study, the binding of phenanthrene to GRSPs might be associated with proteins and H-bonding.

On the other hand, there had been some difference in the Mw fraction of GRSPs bound with phenanthrene, for example, the variation of FTIR spectrogram of the high-Mw fraction (>10,000 Da) complexed with phenanthrene was in accord with the GRSP_*bulk*_ bond to phenanthrene because the GRSP _>__10,000__*Da*_ fraction is a dominant group form of GRSP bulk, while for the <3,000 and 3,000–10,000 Da fractions, the prominent peaks moved slightly (Figures 4A,B), indicating that the interaction between GRSP and phenanthrene is weak. Meanwhile, the hydrophobic forces dominate the interaction process. The above-mentioned results proved that the characteristics of HOCs–GRSP complex have similar features with HOCs–DOM complex. DOM not only acts as H electron donor interacting with the π-base of HOCs but also reacts with itself or other H electron donors as π-base.

### Environmental Implications

In the current study, we found that changes in environmental conditions, such as ion cations and pH values, can affect the phenanthrene interaction with GRSP, especially metal cations. The GRSP complexation with phenanthrene was inhibited with higher metal cations, such as Cu, Fe, and Al, because GRSP may preferentially adsorb large amounts of heavy metals. On the side, the GRSP might agglomerate and sink at high-valence metal cations, weaken the binding capacity, and advance the transfer of PAHs (Gonzalez-Chavez et al., 2004; Vodnik et al., 2008; Wang et al., 2020).

The complexation of contaminants, such as heavy metal, organic pollutants, and nanomaterials, by GRSP strongly alters their fate, i.e., mobility, bioavailability, and toxicity in the environment (Gao et al., 2017; Chen et al., 2018; Wang et al., 2020). On the one hand, GRSP could interact with DOM, enhance PAH dissolution in soil, and further affect the transport of PAHs in the ecosystem (Gao et al., 2017). Meanwhile, it also could improve root PAH accumulation, increasing the weakly and strongly adsorbed fractions of PAHs in roots (Chen et al., 2018). Furthermore, Wang et al. (2020) indicated that GRSP could sequestrate Cu and Cd, form a stable complexation, and promote water quality. However, there are scarce studies concerning the effects of exposure to HOCs associated with GRSP of various molecular weights on both the ecosystem and humans. Rosenstock et al. (2005) found that bacteria preferred DOM in the size fraction <3,000 Da and the non-humic components if available. Moreover, the DOM-associated PAHs were partly bioavailable to organisms, and their bioavailability has effects on the molecular weight of DOM (Lin et al., 2018). The middle-Mw-fraction DOM enhances the bio-absorption of pyrene in water flea through transmembrane transport, although weakening their binding capacity. Moreover, HOCs associated with low-Mw fractions (<3,500 Da) could interact with the plasma membrane of plant cells and be taken up by the plant (Nardi et al., 2002). Hence, for different-Mw GRSP fractions, they could have a similar environmental behavior to DOM, causing the alteration of the fate of organic pollutants and heavy metals in soil, which could bring some risks for the ecological system. Consequently, it is necessary to assess the risk and bioavailability of GRSP_*Mw*_ fraction-bound PAHs in environmental systems in the future.

## Conclusion

Forest soil-extracted GRSPs had a mixture containing protein, polysaccharides, and humic substances, prioritizing 10,000-Da compounds. Different-Mw GRSP fractions have some individuality in the distribution of elements and UV–visible characteristics. H-bound, NH-π reaction, and hydrophobic effect were predominant binding mechanisms for phenanthrene with different-Mw GRSP fractions, particularly the >10,000-Da fraction. Among them, the binding capacity was ordered as follows: F2 < F1 < F3. At the same time, the pH and ion cations in the aquatic system affected the complexation of phenanthrene to GRSP. As an important component of organic matter in soil, GRSPs could sequester PAHs and form complexes; however, they might alter the environmental fate of HOCs in the soil, causing ecological risks due to their different mobility. Our findings provide new insights pertaining to the role of GRSPs in phenanthrene sequestration and the characteristics of GRSP in the spectrum.

## Data Availability Statement

The original contributions presented in the study are included in the article/Supplementary Material, further inquiries can be directed to the corresponding author/s.

## Author Contributions

XZ: conceptualization, methodology, software, data curation, writing-original draft preparation, and manuscript revision. JW: manuscript revising, formal analysis, and writing review and editing. YJ: data curation, visualization, and investigation. GL: investigation and software. GV: language and manuscript revising. MW: language and manuscript revising. YG: conceptualization, supervision, funding acquisition, manuscript revising, and editing. All authors contributed to the article and approved the submitted version.

## Conflict of Interest

The authors declare that the research was conducted in the absence of any commercial or financial relationships that could be construed as a potential conflict of interest.

## Publisher’s Note

All claims expressed in this article are solely those of the authors and do not necessarily represent those of their affiliated organizations, or those of the publisher, the editors and the reviewers. Any product that may be evaluated in this article, or claim that may be made by its manufacturer, is not guaranteed or endorsed by the publisher.
