# Supplementary material for: Characterization of Different Molecular Size Fractions of Glomalin-Related Soil Protein From Forest Soil and Their Interaction With Phenanthrene
Source: Front Microbiol. 2022 Feb 23;12:822831. doi: 10.3389/fmicb.2021.822831 (PMC8905316; doi:10.3389/fmicb.2021.822831)
Supplement: Supplementary file 1 [file Data_Sheet_1.docx]

**Supporting Information for**

**Characterization of different molecular size fractions of glomalin-related soil protein (GRSP) from forest soil and their interaction with phenanthrene**

Xian Zhou^1^, Jian Wang ^1^,Yi Jiang ^1^, Ganghua Leng ^1^, Galina K. Vasilyeva ^2^, Michael Gatheru Waigi^1^, YanzhengGao ^1^*

^1^Institute of Organic Contaminant Control and Soil Remediation, College of Resources and Environmental Sciences, Nanjing Agricultural University, Nanjing 210095, China

^2^ Institute of Physicochemical and Biological Problems in Soil Science, RAS, Pushchino, Moscow region, Russia

*Corresponding author: Yanzheng Gao, Dr/Prof

Tel: +86-2584395194.

E-mail: gaoyanzheng@njau.edu.cn

**Supporting information includes:**

S1. Supplemental materials and methods

S2. Supplemental results

12 pages,5 figures, 5 tables

**S1. Supplemental materials and methods**

**Extraction and fractionation of glomalin relate soil protein (GRSP) by ultrafiltration**

T-GRSP was repeatedly extracted from 4 g soil with 32 mL of 50 mmol/L sodiumcitrate(pH 8.0) at 121 ℃ for 1 h until the supernatant had a straw-colored appearance. For each extraction interval, the supernatant was separated by centrifugation at 10000 rpm (Velocity 18, Dynamica, Germany) for 10 min, combined into a clean flask, and then stored at 4 ℃. Then,as shown in Figure 1, the above supernatants were precipitatedby slowly adding 0.1mol/L HCl until the pH of the solution reached2.0-2.5. The acidified supernatants were incubated in ice for 60 min,followed by centrifugation at 10000rpm for 10 min. The precipitate wasthen re-dissolved in 0.1 mol/L NaOH, filtered with 0.45 μm membrane to remove particulate matter, and the prepared solutions of GRSP were stored at 4 ℃.

Then the GRSP solution was fractionated into three fractions with various molecular weights by a Millipore ultrafiltration tube, equipped with ultrafiltration membranes with nominal molecular weight cutoffs of 10 000 and 3 000 Da. Specifically, ultrafiltration membranes should be rinsed with ultrapure water at <4000×g centrifuge for 10-40 min. The tube was used 0.01 mol/L NaOH solution and then rinsed with ultrapure water to eliminate the organic carbon on the membrane that originated from the membrane and the residue of before. Then, 15 mL GRSP stocked solution was added in an ultrafiltration tube centrifuging at 4000×g for 15 min. Afterward, the solution in the inner tube was drained carefully using a pipette gun, and the liquids in the outer tube were moved to another tube. The ultrafiltration experiment was conducted from high molecular weight (10 000 Da) to low molecular weight (3 000 Da) to reduce the influence of concentration polarization of macromolecular organic matter on the ultrafiltration procedure. The different GRSP fractions received from the ultrafiltration experiment mentioned above were F1 (<3 000 Da), F2 (3 000-10 000 Da), F3 (> 10 000 Da), and the unfractionated GRSP was expressed as FU. The photograph of F1-F3 and FU solutions is shown in Figure S1. The color of GRSP solution with various molecular weights became darker with the increasing molecular weight of GRSP, which was in accordance with the other DOM research (Lin et al., 2018). All GRSP fractions were freeze-dried by lyophilizer(ALPHA 2-4 LD plus, Christ, Germany) to be stored at a vacuum dryer.

**Characterization of GRSP with various molecular weights**

The molecular weight of GRSP (2 mg/mL) was characterized bygel permeation chromatography (GPC) (Waters1525, USA). And other instrumental conditions were as follows:And other instrumental conditions were as follows: TSK gel:46 G3000PWXL, Column No.: S0127, Detector: differential refraction detector, Temperature: 30 °C. As shown in Figure S2, the GPC graph of the GRSP fractions was depicted. The results found that the distribution of the molecular weight of GRSP was wide, especially >10 000 Da fraction, which was accounted for with 63% (Table S2, Figure S2).At the same time, in order to determine the stability of Ultrafiltration Centrifugal Tube, the molecular sizes of other GRSP fractions were determined, and they are 1,209 (F1), 4209 (F2), and 20,641Da (F3), respectively.

**Characterization of GRSP with molecular diversity**

Extraction protocol and procedure for SOM molecular assay followed Chen et al. (2021). The protocol involved solvent extraction, derivatization, and GC/MS quantification. The detailed extraction procedure was as follows:

1 g freeze-dried GRSP was added in a glass flask and refluxed at 100 °C for 3 h with 30 ml of 1 M methanolic KOH. The extract was acidified to pH 1 with 12 M HCl, and the solvents were then removed with rotary evaporation. Lipids were recovered from the water phase by liquid-liquid extraction with diethyl ether, concentrated again viarotary evaporation, and dried under nitrogen gas.For molecular identification, the extracts obtained above were recovered with 1 mL HPLC dichloromethane/methanol (1:1, v/v) and passed through an organic needle filter of 0.22 μm.The solution was then transferred to a 1.5 mL vial and added the internal standard solutionsof deuterated pyridine (C_24_D_50_)solution. Each mixed solution was concentrated in a solvent evaporating system (Genevac® EZ-2 plus) and reacted with N, O-bis-(trimethylsilyl) trifluoroacetamide (BSTFA), and pyridine (9:1, v/v) for 3 h at 70 °C to obtain trimethylsilyl (TMS) derivatives. After cooling, 900 μL n-hexane (HPLC) was added to each vial for diluting the solution to 1 mL prior to GC/MS (Agilent, USA) detection.

The GC analysis was operated in this condition: temperature held at 65 ℃for 2 min and increased from 65 to 290 ℃, and a final isothermal hold at 290 ℃ for 20 min. Then MS analysis was worked at 70 eV ionization energy and scanned from 50 to 650 Da. Finally, the data was obtained and processed in the Agilent Mass Hunter Workstation.The information on organic compounds in GRSP was acquired in Table S3.

The molecular abundance (MA) was calculated with an equation:

$MA=\frac{V_{i}\times C_{i}\times S_{e}}{M_{s}\times C_{s}\times S_{i}}$ (1)

Where *V_i_* is the volume of internal standard added in the vial, pyridineconcentration is 1.05 g/mL (*C_i_*); *S_i_* and *S_e_*are the compound peak areas of the internal standard and the GRSP extract, respectively; *Ms* is the mass weight of GRSP.

**Effectsof environmental conditions on the interaction between phenanthreneand GRSP**

Excitation wavelength (Ex, nm)/emission wavelength (Em, nm) was set at 292/365 nm (phenanthrene), the pH influence (3.0-11.0) of the phenanthrene-GRSPs binding isotherm was recorded at T=25 ℃ and I=2 mmol/L; the ionic strength variation (0-200 mmol/L) of the phenanthrene-GRSPs interaction was measured at T=25 ℃ and pH=7.0; the metal cation types, including K^+^, Na^+^, Ca^2+^, Mg^2+^, Cu^2+^, Al^3+^ were investigated at 25℃ and pH=7.0.

**Thermodynamics of interaction between phenanthreneand GRSP**

The magnitudes and signs of thermodynamic functions could reflect the phe stability for binding reactions (Zhao et al., 2019). Hence, the thermodynamics of phe-GRSP interaction for three temperatures (25, 35, 45 ℃) was investigated at pH=7.0 and I=2 mmol/L. If △H^0^ does not fluctuate substantially throughout the temperature extent analyzed, its value and the △H^0^ can be concluded from Van’t Hoff equation as Eq. (2):

$\ln K=\frac{-\Delta H^{0}}{RT}+\frac{\Delta S^{0}}{R}$ (2)

where T is absolute temperature, R is the ideal gas constant, and K is the binding constant at the corresponding temperature. Furtherly, the △G^0^ is assessed from the equation as Eq. (3):

$\Delta G^{0}=\Delta H^{0}-T\Delta S^{0}$ (3)

**S2. Supplemental results**

**Table S1.**The physical characteristics of Purple Mountain forest soil

| **Soil** | **pH** | **SOC(g/kg)** | **CEC cmol (+)/kg** | **Sand(%)** | **Clay(%)** | **Silt(%)** |
| --- | --- | --- | --- | --- | --- | --- |
| Yellow Brown Soil | 6.63 | 51.1 | 25.7 | 18.59 | 64.96 | 16.44 |

**Table S2.** Relative molecular weight distribution of GRSP

| No. | Slice *Mw* (Daltons) | Slice  Log *Mw* | Slice Volume (mL) | Slice Area | dwt/d(log M) | Cumulative (%) |
| --- | --- | --- | --- | --- | --- | --- |
| 1 | 88311 | 4.95 | 7.42 | 798 | 0.097 | 1.00 |
| 2 | 74525 | 4.87 | 7.49 | 1338 | 0.164 | 2.00 |
| 3 | 66414 | 4.82 | 7.55 | 1792 | 0.222 | 3.00 |
| 4 | 60684 | 4.78 | 7.59 | 2189 | 0.272 | 4.00 |
| 5 | 56250 | 4.75 | 7.62 | 2540 | 0.317 | 5.00 |
| 6 | 52633 | 4.72 | 7.65 | 2862 | 0.359 | 6.00 |
| 7 | 49595 | 4.70 | 7.68 | 3161 | 0.398 | 7.00 |
| 8 | 46980 | 4.67 | 7.71 | 3435 | 0.434 | 8.00 |
| 9 | 44682 | 4.65 | 7.73 | 3692 | 0.468 | 9.00 |
| 10 | 42635 | 4.63 | 7.75 | 3936 | 0.500 | 10.00 |
| 11 | 40805 | 4.61 | 7.77 | 4165 | 0.530 | 11.00 |
| 12 | 39142 | 4.59 | 7.79 | 4383 | 0.559 | 12.00 |
| 13 | 37623 | 4.57 | 7.81 | 4593 | 0.587 | 13.00 |
| 14 | 36226 | 4.56 | 7.82 | 4794 | 0.614 | 14.00 |
| 15 | 34937 | 4.54 | 7.84 | 4988 | 0.640 | 15.00 |
| 16 | 33741 | 4.53 | 7.86 | 5176 | 0.666 | 16.00 |
| 17 | 32628 | 4.51 | 7.88 | 5358 | 0.690 | 17.00 |
| 18 | 31588 | 4.50 | 7.89 | 5537 | 0.715 | 18.00 |
| 19 | 30612 | 4.49 | 7.91 | 5709 | 0.738 | 19.00 |
| 20 | 29695 | 4.47 | 7.92 | 5875 | 0.761 | 20.00 |
| 21 | 28829 | 4.46 | 7.94 | 6036 | 0.783 | 21.00 |
| 22 | 28010 | 4.45 | 7.95 | 6190 | 0.804 | 22.00 |
| 23 | 27237 | 4.43 | 7.96 | 6335 | 0.824 | 23.00 |
| 24 | 26502 | 4.42 | 7.98 | 6475 | 0.844 | 24.00 |
| 25 | 25800 | 4.41 | 7.99 | 6607 | 0.862 | 25.00 |
| 26 | 25129 | 4.40 | 8.001 | 6730 | 0.879 | 26.00 |
| 27 | 24489 | 4.39 | 8.014 | 6848 | 0.896 | 27.00 |
| 28 | 23876 | 4.38 | 8.026 | 6953 | 0.911 | 28.00 |
| 29 | 23286 | 4.37 | 8.038 | 7051 | 0.925 | 29.00 |
| 30 | 22718 | 4.36 | 8.050 | 7144 | 0.938 | 30.00 |
| 31 | 22172 | 4.35 | 8.062 | 7225 | 0.950 | 31.00 |
| 32 | 21645 | 4.33 | 8.073 | 7299 | 0.961 | 32.00 |
| 33 | 21134 | 4.32 | 8.085 | 7365 | 0.971 | 33.00 |
| 34 | 20641 | 4.31 | 8.096 | 7426 | 0.980 | 34.00 |
| 35 | 20163 | 4.30 | 8.108 | 7477 | 0.988 | 35.00 |
| 36 | 19699 | 4.29 | 8.119 | 7521 | 0.995 | 36.00 |
| 37 | 19248 | 4.28 | 8.130 | 7559 | 1.000 | 37.00 |
| 38 | 18810 | 4.27 | 8.142 | 7588 | 1.006 | 38.00 |
| 39 | 18383 | 4.26 | 8.153 | 7614 | 1.010 | 39.00 |
| 40 | 17968 | 4.25 | 8.164 | 7632 | 1.014 | 40.00 |
| 41 | 17563 | 4.24 | 8.175 | 7642 | 1.016 | 41.00 |
| 42 | 17167 | 4.23 | 8.186 | 7648 | 1.018 | 42.00 |
| 43 | 16781 | 4.22 | 8.197 | 7644 | 1.019 | 43.00 |
| 44 | 16403 | 4.21 | 8.209 | 7632 | 1.018 | 44.00 |
| 45 | 16033 | 4.20 | 8.220 | 7616 | 1.017 | 45.00 |
| 46 | 15671 | 4.19 | 8.231 | 7594 | 1.015 | 46.00 |
| 47 | 15315 | 4.19 | 8.242 | 7560 | 1.012 | 47.00 |
| 48 | 14966 | 4.18 | 8.254 | 7520 | 1.007 | 48.00 |
| 49 | 14623 | 4.16 | 8.265 | 7471 | 1.001 | 49.00 |
| 50 | 14286 | 4.15 | 8.277 | 7414 | 0.995 | 50.00 |
| 51 | 13953 | 4.14 | 8.288 | 7348 | 0.987 | 51.00 |
| 52 | 13627 | 4.13 | 8.300 | 7275 | 0.979 | 52.00 |
| 53 | 13303 | 4.12 | 8.312 | 7191 | 0.968 | 53.00 |
| 54 | 12983 | 4.11 | 8.324 | 7099 | 0.957 | 54.00 |
| 55 | 12668 | 4.10 | 8.336 | 7001 | 0.945 | 55.00 |
| 56 | 12356 | 4.09 | 8.349 | 6896 | 0.931 | 56.00 |
| 57 | 12046 | 4.08 | 8.361 | 6782 | 0.917 | 57.00 |
| 58 | 11740 | 4.07 | 8.374 | 6664 | 0.902 | 58.00 |
| 59 | 11436 | 4.06 | 8.387 | 6541 | 0.886 | 59.00 |
| 60 | 11135 | 4.05 | 8.400 | 6413 | 0.870 | 60.00 |
| 61 | 10836 | 4.03 | 8.414 | 6286 | 0.854 | 61.00 |
| 62 | 10539 | 4.02 | 8.428 | 6159 | 0.837 | 62.00 |
| 63 | 10244 | 4.01 | 8.442 | 6031 | 0.821 | 63.00 |
| 64 | 9953 | 4.00 | 8.456 | 5904 | 0.804 | 64.00 |
| 65 | 9664 | 3.98 | 8.471 | 5781 | 0.789 | 65.00 |
| 66 | 9379 | 3.97 | 8.486 | 5662 | 0.773 | 66.00 |
| 67 | 9096 | 3.96 | 8.502 | 5545 | 0.758 | 67.00 |
| 68 | 8817 | 3.94 | 8.517 | 5430 | 0.743 | 68.00 |
| 69 | 8541 | 3.93 | 8.533 | 5321 | 0.729 | 69.00 |
| 70 | 8268 | 3.92 | 8.550 | 5209 | 0.715 | 70.00 |
| 71 | 7999 | 3.90 | 8.566 | 5095 | 0.700 | 71.00 |
| 72 | 7732 | 3.89 | 8.584 | 4978 | 0.685 | 72.00 |
| 73 | 7469 | 3.87 | 8.601 | 4854 | 0.668 | 73.00 |
| 74 | 7208 | 3.86 | 8.619 | 4725 | 0.651 | 74.00 |
| 75 | 6948 | 3.84 | 8.638 | 4586 | 0.633 | 75.00 |
| 76 | 6691 | 3.82 | 8.657 | 4434 | 0.613 | 76.00 |
| 77 | 6434 | 3.81 | 8.677 | 4276 | 0.592 | 77.00 |
| 78 | 6179 | 3.79 | 8.697 | 4108 | 0.569 | 78.00 |
| 79 | 5923 | 3.77 | 8.719 | 3932 | 0.545 | 79.00 |
| 80 | 5667 | 3.75 | 8.742 | 3757 | 0.522 | 80.00 |
| 81 | 5411 | 3.73 | 8.765 | 3588 | 0.499 | 81.00 |
| 82 | 5156 | 3.71 | 8.790 | 3429 | 0.478 | 82.00 |
| 83 | 4904 | 3.69 | 8.816 | 3284 | 0.458 | 83.00 |
| 84 | 4655 | 3.67 | 8.842 | 3464 | 0.442 | 84.00 |
| 85 | 4411 | 3.64 | 8.870 | 3057 | 0.427 | 85.00 |
| 86 | 4173 | 3.62 | 8.899 | 2968 | 0.416 | 86.00 |
| 87 | 3941 | 3.60 | 8.928 | 2886 | 0.405 | 87.00 |
| 88 | 3718 | 3.57 | 8.958 | 2813 | 0.395 | 88.00 |
| 89 | 3502 | 3.54 | 8.989 | 2737 | 0.385 | 89.00 |
| 90 | 3292 | 3.52 | 9.021 | 2653 | 0.373 | 90.00 |
| 91 | 3089 | 3.49 | 9.054 | 2561 | 0.361 | 91.00 |
| 92 | 2891 | 3.46 | 9.088 | 2453 | 0.346 | 92.00 |
| 93 | 2697 | 3.43 | 9.124 | 2332 | 0.329 | 93.00 |
| 94 | 2506 | 3.40 | 9.163 | 2193 | 0.310 | 94.00 |
| 95 | 2316 | 3.36 | 9.204 | 2030 | 0.287 | 95.00 |
| 96 | 2125 | 3.33 | 9.248 | 1841 | 0.261 | 96.00 |
| 97 | 1929 | 3.29 | 9.299 | 1611 | 0.228 | 97.00 |
| 98 | 1721 | 3.24 | 9.358 | 1325 | 0.188 | 98.00 |
| 99 | 1479 | 3.17 | 9.437 | 919 | 0.130 | 99.00 |
| 100 | 923 | 2.97 | 9.683 | 20 | 0.003 | 100.00 |

**Table S3.** The mainmolecules of GRSP identified with GC/MS and the assigned functional groups with reference to NIST library 2017

| No. | CAS | Formula | m/z | Group | Abundance |
| --- | --- | --- | --- | --- | --- |
| 1 | 141-43-6 | C2H7NO | 61.08 | AM | 750.6 |
| 2 | 51-61-6 | C8H11NO2 | 153.18 | AM | 21.45 |
| 3 | 17016-13-6 | C2H4N2O2 | 88.07 | AM | 15.39 |
| 4 | 112-30-1 | C10H22O | 158.29 | AM | 4.27 |
| 5 | 27073-41-2 | C6H6O | 94.11 | PH | 5.89 |
| 6 | 121-34-6 | C8H8O4 | 168.15 | PH | 6.39 |
| 7 | 1000362-63-1 | C7H7N5O | 177.16 | PH | 1.88 |
| 8 | 50-21-5 | C3H6O3 | 90.088 | HA | 5.91 |
| 9 | 26124-68-5 | C2H4O3 | 76.05 | HA | 7.41 |
| 10 | 77-92-9 | C6H8O7 | 192.12 | HA | 1.31 |
| 11 | 463-77-4 | CH3NO2 | 89.09 | HA | 4.05 |
| 12 | 14047-56-4 | C4H6O4 | 118.09 | FA | 4.19 |
| 13 | 124-04-9 | C6H10O4 | 146.14 | FA | 5.22 |
| 14 | 654-15-9 | C9H9N3O2 | 191.87 | FA | 1.06 |
| 15 | 287399-29-5 | C7H6O3 | 144.08 | FA | 4.75 |
| 16 | 64118-37-2 | C5H10O2 | 102.13 | FA | 3.12 |
| 17 | 505-48-6 | C8H14O4 | 174.19 | FA | 2.19 |
| 18 | 123-99-9 | C9H16O4 | 188.22 | FA | 5.22 |
| 19 | 544-63-8 | C14H28O2 | 228.37 | FA | 3.04 |
| 20 | 57-10-3 | C16H32O2 | 256.42 | FA | 2.45 |
| 21 | 506-12-7 | C17H34O2 | 270.45 | FA | 1.17 |
| 22 | 112-80-1 | C18H34O2 | 282.46 | FA | 26.72 |
| 23 | 1957-11-04 | C17H36O2 | 254.41 | FA | 13.21 |
| 24 | 1120-28-1 | C21H42O2 | 326.56 | FA | 9.76 |
| 25 | 373-49-9 | C16H30O2 | 254.41 | FA | 5.34 |
| 26 | 929-77-1 | C23H46O2 | 354.61 | FA | 6.54 |
| 27 | 1000315-48-6 | C17H24O4 | 292.37 | FA | 10.68 |
| 28 | 65-85-0 | C7H6O2 | 122.12 | BA | 3.34 |
| 29 | 23395-75-7 | C11H10N2O3 | 218.21 | AD | 11.11 |
| 30 | 29122-68-7 | C14H22N2O3 | 266.34 | AD | 10.56 |
| 31 | 75-12-7 | CH3NO | 45.04 | AD | 4.30 |
| 32 | 52089-32-4 | C10H14O | 150.22 | AC | 1.30 |
| 33 | 14251-57-1 | C15H12 | 192 | HC | 3.28 |
| 34 | 112-43-6 | C11H22O | 170.29 | HC | 1.63 |
| 35 | 100-21-0 | C8H6O4 | 166.13 | BE | 5.44 |
| 36 | 52476-90-1 | C4H5N7 | 235 | ON | 1.14 |
| 37 | 284474-48-2 | C7H6O2 | 122.12 | KE | 1.18 |
| 38 | 99-76-3 | C8H8O3 | 152.15 | ES | 2.71 |
| 39 | 1587-20-8 | C9H14O7 | 234.20 | ES | 2.30 |
| 40 | 1679-64-7 | C9H8O4 | 180.16 | ES | 2.07 |
| 41 | 3943-74-6 | C9H10O4 | 182.17 | ES | 3.44 |
| 42 | 5129-58-8 | C15H30O2 | 242.40 | ES | 3.21 |
| 43 | 1000424-50-7 | C16H32O2 | 256.42 | ES | 6.54 |
| 44 | 112-39-0 | C17H34O2 | 270.45 | ES | 62.01 |
| 45 | 6929-04-0 | C18H36O2 | 284.48 | ES | 3.68 |
| 46 | 2490-49-5 | C18H36O2 | 284.48 | ES | 4.96 |
| 47 | 5802-82-4 | C27H54O2 | 410.72 | ES | 3.96 |
| 48 | 3076-04-8 | C16H30O2 | 284.48 | ES | 1.98 |

Note: AC: Alcohol; AD: Amides; AM: Amines; PH: Polyphenols; HA: Hydroxyl acids; FA: Fatty acids; BA: Benzoic acids; HC: others hydrogen carbons; BE: Benzoates; ON: Organic nitrogens; KE: Ketones; ES: Aliphatic esters.

**Table S4.** Binding parameters and thermodynamic parameters of GRSP-phenanthrene.

| System | T (K) | Site-binding equation | |  | △G^0^ (KJ/mol) | △S^0^ (J/mol·K) | △H^0^ (KJ/mol) | Stern-Volmer equation | |
| --- | --- | --- | --- | --- | --- | --- | --- | --- | --- |
|  |  | n | K_A_(L/kg) | R^2^ |  |  |  | Ksv (L/kg) | R^2^ |
| GRSP_<3000_-phe | 298 | 1.1964 | 425.3 | 0.9795 | -14.48 | 302.5 | 75.67 | 1000 | 0.9975 |
|  | 308 | 1.0141 | 608 | 0.9921 | -17.51 |  |  | 700 | 0.9979 |
|  | 318 | 0.7652 | 2944 | 0.9496 | -20.54 |  |  | 1000 | 0.9852 |
| GRSP_3000-10000_-phe | 298 | 1.1445 | 827 | 0.9692 | -16.65 | 88.57 | 9.74 | 1500 | 0.9968 |
|  | 308 | 1.1478 | 944 | 0.9962 | -17.53 |  |  | 1700 | 0.9917 |
|  | 318 | 1.1885 | 1059 | 0.9599 | -18.42 |  |  | 2300 | 0.9075 |
| GRSP_>10000_-phe | 298 | 1.2961 | 3481 | 0.9988 | -20.39 | 177.3 | 32.46 | 13700 | 0.9914 |
|  | 308 | 1.138 | 6667 | 0.9977 | -54.62 |  |  | 13600 | 0.9913 |
|  | 318 | 1.0976 | 7896 | 0.995 | -56.39 |  |  | 13600 | 0.9889 |
| GRSP-phe | 298 | 1.1386 | 8106 | 0.9879 | -22.22 | 52.24 | -6.661 | 11900 | 0.9339 |
|  | 308 | 1.1619 | 6808 | 0.995 | -22.75 |  |  | 15700 | 0.9032 |
|  | 318 | 1.184 | 6858 | 0.9928 | -23.27 |  |  | 18200 | 0.9799 |

**TableS5.** Effect of metal cation typeon the interaction between GRSP and phenanthrene.

| Fractions | K_GRSP_（L/mg） | | | | | | |
| --- | --- | --- | --- | --- | --- | --- | --- |
|  | CK | K^+^ | Na^+^ | Mg^2+^ | Ca^2+^ | Cu^2+^ | Al^3+^ |
| F1 | 0.12±0 | 0.27±0 | 0.29±0.01 | 0.19±0.01 | 0.14±0 | 0.25±0.01 | 0.1±0.01 |
| F2 | 0.07±0 | 0.08±0.01 | 0.08±0 | 0.08±0 | 0.06±0 | 0.1±0.01 | 0.01±0.02 |
| F3 | 0.5±0.02 | 0.51±0.03 | 0.46±0.01 | 0.48±0.02 | 0.35±0.03 | 0.12±0 | 0.1±0 |
| FU | 0.31±0.01 | 0.35±0 | 0.36±0 | 0.37±0.01 | 0.26±0 | 0.24±0.01 | 0.12±0.01 |


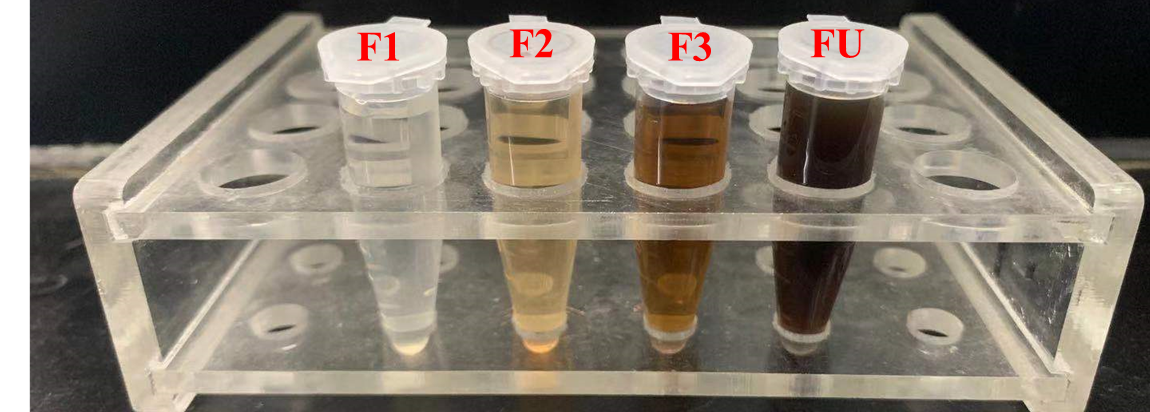


**Figure S1.** Samples of dialyzed GRSP and four fractions of GRSP through the dialysis membrane (F1: <3,000 Da, F2: 3,000-10,000 Da,F3: >10,000 Da,FU: unfractionated GRSP).


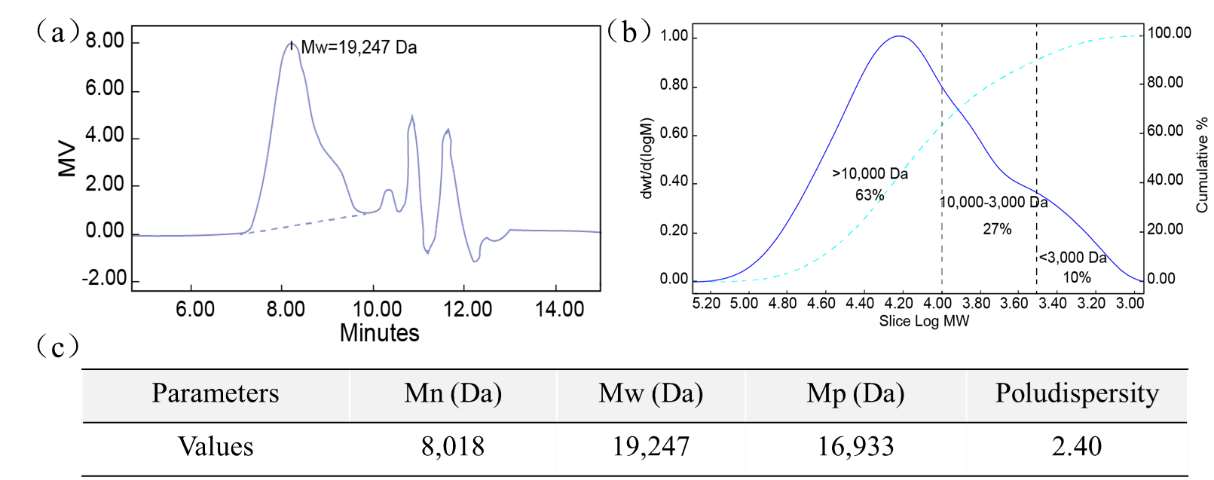


**Figure S2.** Gel permeation chromatography graph of the GRSP (2 mg/mL) (a),the molecular weight distribution of GRSP (b), and broad GRSP relative peak as shown in (c).


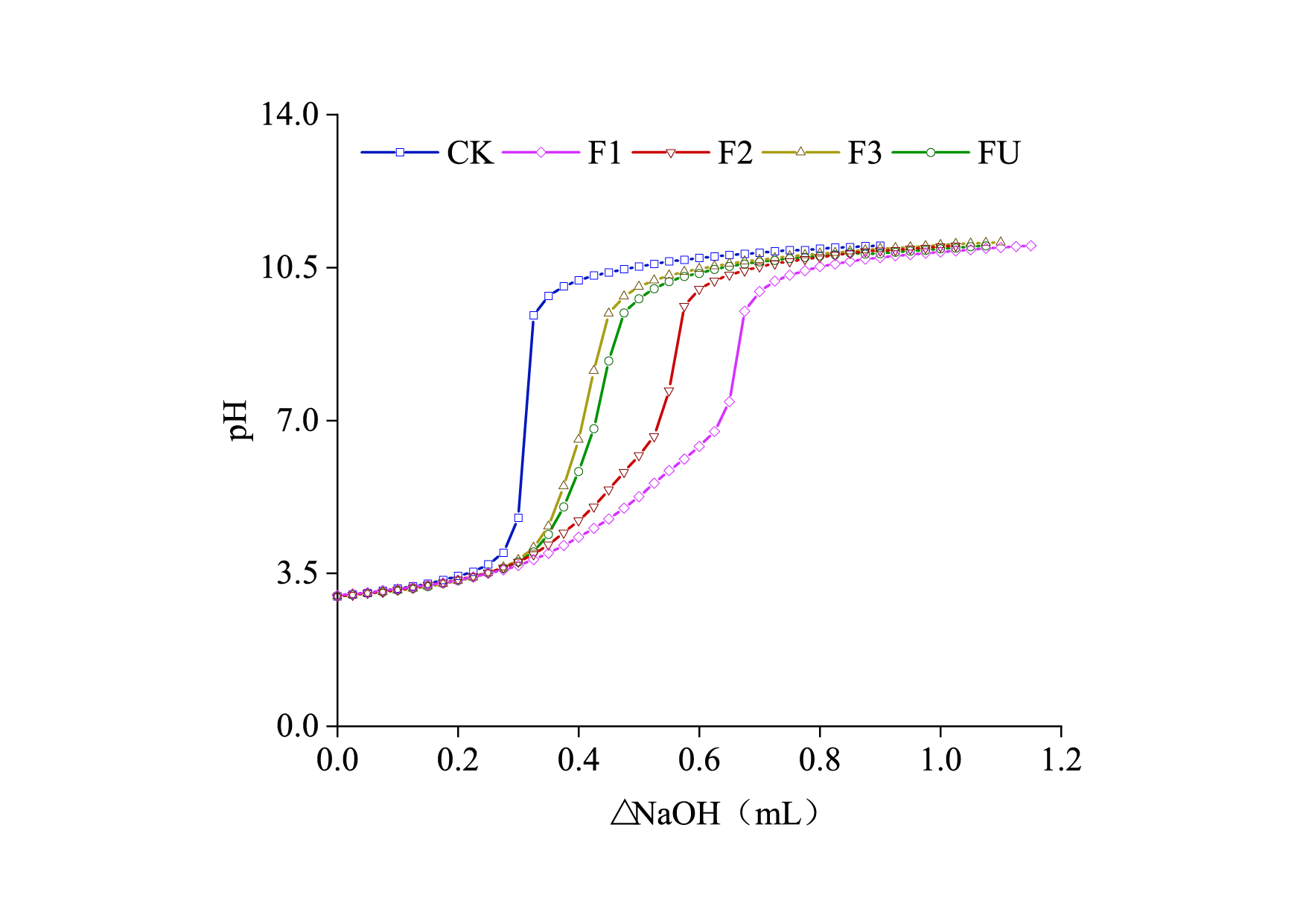


**Figure S3**. The curve of potentiometric titration of GRSP and its MW fraction


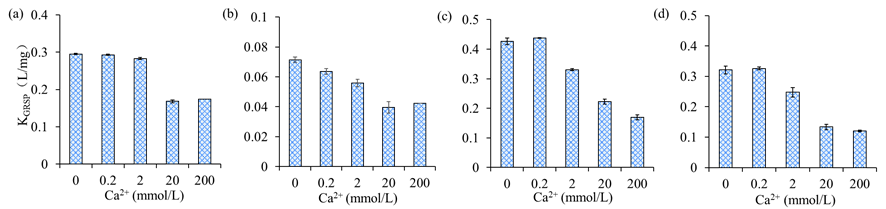


**Figure S4.** Effect of ionic strengths on the interaction between GRSP and phenanthrene.


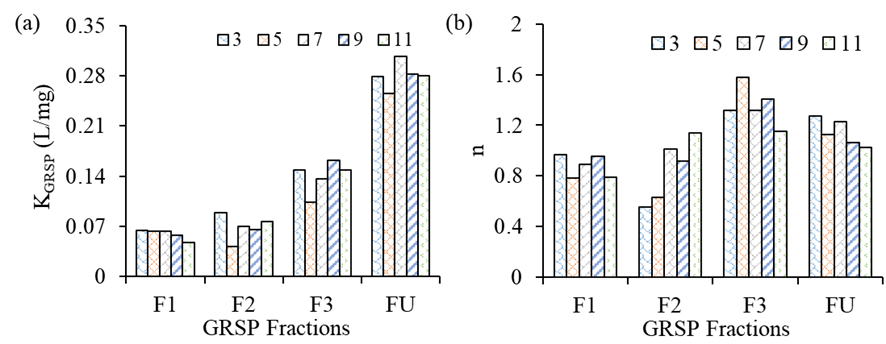


**Figure S5**. Effectof pH on the interaction between GRSP and phenanthrene.

**Reference**

Chen, S., Ding, Y., Xia, X., Feng, X., Liu, X., Zhang, J., Drosos, M., Cheng, K., Bian, R., Zhang, X., Li, L., Pan, G., 2021. Amendment of straw biochar increased molecular diversity and enhanced preservation of plant derived organic matter in extracted fractions of a rice paddy. J. Environ Manage. 285, 112104.

Lin, H., Xia, X., Bi, S., Jiang, X., Wang, H., Zhai, Y., Wen, W. 2018. Quantifying bioavailability of pyrene associated with dissolved organic matter of various molecular weights to *Daphnia magna*. Environ. Sci. Technol. 52, 644−653.

Zhao, X., Hu, Z., Yang, X., Cai, X., Wang, Z., Xie, X. 2019. Noncovalent interactions between fluoroquinolone antibiotics with dissolved organic matter: A ^1^H NMR binding site study and multi-spectroscopic methods. Environ Pollut. 248, 815−822.
